# Behavioral and psychological impact of genome sequencing: a pilot randomized trial of primary care and cardiology patients

**DOI:** 10.1038/s41525-021-00236-2

**Published:** 2021-08-24

**Authors:** Kurt D. Christensen, Erica F. Schonman, Jill O. Robinson, J. Scott Roberts, Pamela M. Diamond, Kaitlyn B. Lee, Robert C. Green, Amy L. McGuire

**Affiliations:** 1 PRecisiOn Medicine Translational Research (PROMoTeR) Center, Department of Population Medicine, Harvard Pilgrim Health Care Institute, Boston, MA USA; 2 Department of Population Medicine, Harvard Medical School, Boston, MA USA; 3 Broad Institute of Harvard and MIT, Cambridge, MA USA; 4 Division of Genetics, Department of Medicine, Brigham and Women’s Hospital, Boston, MA USA; 5 Center for Medical Ethics and Health Policy at Baylor College of Medicine, Houston, TX USA; 6 Department of Health Behavior and Health Education, University of Michigan School of Public Health, Ann Arbor, MI USA; 7 Center for Health Promotion and Prevention Research, University of Texas Houston School of Public Health, Houston, TX USA; 8 Partners Personalized Medicine, Boston, MA USA; 9 Department of Medicine, Harvard Medical School, Boston, MA USA; 10 Ariadne Labs, Boston, MA USA

**Keywords:** Translational research, Molecular medicine, Lifestyle modification, Predictive markers, Genetics research

## Abstract

Many expect genome sequencing (GS) to become routine in patient care and preventive medicine, but uncertainties remain about its ability to motivate participants to improve health behaviors and the psychological impact of disclosing results. In a pilot trial with exploratory analyses, we randomized 100 apparently healthy, primary-care participants and 100 cardiology participants to receive a review of their family histories of disease, either alone or in addition to GS analyses. GS results included polygenic risk information for eight cardiometabolic conditions. Overall, no differences were observed between the percentage of participants in the GS and control arms, who reported changes to health behaviors such as diet and exercise at 6 months post disclosure (48% vs. 36%, respectively, *p* = 0.104). In the GS arm, however, the odds of reporting a behavior change increased by 52% per high-risk polygenic prediction (*p* = 0.032). Mean anxiety and depression scores for GS and control arms had confidence intervals within equivalence margins of ±1.5. Mediation analyses suggested an indirect impact of GS on health behaviors by causing positive psychological responses (*p* ≤ 0.001). Findings suggest that GS did not distress participants. Future research on GS in more diverse populations is needed to confirm that it does not raise risks for psychological harms and to confirm the ability of polygenic risk predictions to motivate preventive behaviors.

## Introduction

Many experts expect genome sequencing (GS) to become a routine part of patient care^1,2^ and advocates envision a future where GS is used for screening purposes to identify predispositions for disease and facilitate targeted prevention^3^. Others urge caution given the potential for patients and providers to be upset and confused by GS results^4–6^. Empirical evidence to inform discussions about the benefits and harms of clinical use of GS is currently lacking, including for behavioral and psychological outcomes^7^.

Extensive research on single-gene and panel genetic testing consistently show that identification of highly penetrant variants for modifiable conditions sometimes improves screening compliance, but sustained changes in lifestyle behaviors, such as physical activity and diet, are rare in at-risk and sick populations^8–12^. Numerous studies have shown that negative psychological responses are also rare, even when findings indicate increased risks for conditions lacking proven preventive options^10–17^. However, there are reasons to believe that individuals may respond differently to GS^18^. GS can provide insight about any condition with established gene–disease associations, whereas single-gene and panel tests have traditionally been used to examine a predefined set of conditions. Results can also be unexpected and prior work has shown behavioral and psychological benefits to disclosing unanticipated secondary findings from single-gene testing^14^. Moreover, GS can identify many types of risk factors, from monogenic disease risks for rare conditions to polygenic risk predictions for common diseases, to carrier status for autosomal recessive conditions. Consequently, behavioral and psychological outcomes have been a priority of research networks such as the Clinical Sequencing Exploratory Research (CSER) Consortium^19^.

In this study, we analyzed data from the MedSeq Project, a pilot randomized trial of GS in cardiology and primary care. A prior descriptive summary of the primary-care cohort found new monogenic findings in 11 patients, two of whom had evidence of phenotypes predicted by those results. It also showed similar scores on scales of anxiety and depression at 6 months post disclosure and rates of self-reported health behavior changes that may have been higher following sequencing (41% of patients receiving GS vs. 30% of patients who did not)^20^. A meta-analysis that combined MedSeq data with data from other CSER sequencing sites showed decreases in anxiety and depression scores at 6 weeks post disclosure among patients who received GS^21^.

Here we expand upon those analyses by reporting findings from the MedSeq Project, one of the first randomized studies of GS, and compared behavioral and psychological outcomes in patient-participants who received GS and family history (FH) reviews vs. patient-participants who only received FH reviews. A goal of this pilot project was to “generate novel hypotheses to inform the design of larger studies moving forward”^22^. However, we also proposed the following exploratory hypotheses a priori: (1) patients who received GS would be more likely to report a change to a health behavior than patients who did not and (2) patients who received GS would show equivalent levels of general anxiety and depression as patients who did not. We summarize findings from those analyses here as well as summarizing physician recommendations for behavior changes because of the important role physicians play in facilitating lifestyle modifications^23,24^. We also summarize the impact of GS on psychological outcomes that included test-related distress, uncertainty, and positive impact, as well as affective responses to information of worry, happiness, concern, empowerment, disappointment, and relief. Finally, we examined whether certain psychological responses may have influenced whether or not GS led to health behavior changes.

## Results

### Participant characteristics and genomic findings

Two hundred and four patient-participants were randomized in the study and 200 completed results disclosure sessions (Supplementary Fig. 1). Patient-participants were primarily non-Hispanic White, with most reporting a household income exceeding $100,000 and a college-level education or higher (Table 1). Physician-participants who disclosed results included seven cardiologists and nine primary-care physicians who reviewed FH information and GS findings, if applicable, with between 2 and 28 patient-participants each (mean: 12.5). As noted previously^20,25–27^, 24 of 49 (49%) cardiology patient-participants in the GS arm received findings related to their hypertrophic or dilated cardiomyopathy (HCM or DCM) diagnosis, including 1 patient whose prior panel testing was negative and 3 patient-participants who had a second variant of uncertain significance identified in addition to a variant of uncertain significance that had been identified previously. In addition, 8 cardiology patient-participants (16%) were identified with a secondary finding associated with a monogenic condition and 41 (84%) were identified with carrier status for at least 1 autosomal recessive condition. Among the 50 primary-care patient-participants randomized to GS, 13 (26%) were identified with a monogenic disease risk, including 2 patient-participants identified with variants associated with hemochromatosis who had been diagnosed with the condition previously. All 50 sequenced primary-care patient-participants (100%) were identified with carrier status for at least 1 autosomal recessive condition. As detailed in the “Methods” section, all patient-participants randomized to GS also received pharmacogenomic information about five common medications, as well as risk information about eight cardiometabolic traits.

**Table 1:** Participant characteristics.

Characteristic *n* (%) unless noted	Cardiology (*n* = 100)	Primary care (*n* = 100)
Mean age (SD)	55.9 (14.2)	54.8 (7.3)
Age range	18.7–84.6	41.2–67.9
Gender		
Female	43 (43%)	58 (58%)
Male	57 (57%)	42 (42%)
Race		
Non-Hispanic White	88 (88%)	87 (87%)
Other	12 (12%)	13 (13%)
Annual Household Income		
<$100,000	43 (43%)	25 (26%)
≥$100,000	53 (53%)	71 (74%)
No response	4 (4%)	4 (4%)
Education		
Did not graduate from college	22 (22%)	14 (14%)
College graduate or higher	78 (78%)	86 (86%)
Has health insurance	100 (100%)	98 (98%)
Diagnosis (cardiology cohort)		
Hypertrophic cardiomyopathy	79 (79%)	
Dilated cardiomyopathy	21 (21%)	

### Behavioral impact

Physician-participants were more likely to recommend lifestyle or medication changes to patient-participants in the GS arm compared to patient-participants in the control arm (23% vs. 12%, respectively; *p* = 0.036). In addition, cardiologists were more likely to recommend lifestyle or medication changes than primary-care physicians (22% vs. 9%, respectively; *p* = 0.002). However, all analyses of patient-reported behavioral outcomes were not statistically significant in analyses that compared randomization arms (all *p* > 0.06). At 6 months post disclosure, 48% of patient-participants in the GS arm and 36% of patient-participants in the control arm reported a change to at least one health behavior (*p* = 0.104, Table 2). Randomization status was also not statistically significant in cohort-specific analyses (Supplementary Table 1).

**Table 2:** Percentage of providers reporting health behavior change recommendations or patients reporting health behavior changes.

Time point/behavior	Control (*n* = 101)	GS (*n* = 99)	OR (95% CI)	*p*
Provider recommendations				
Any change	12%	23%	2.3 (1.1–5.2)	0.036
Health behavior	12%	19%	1.8 (0.8–4.0)	0.157
Medication	2%	7%	3.9 (0.9–27.2)	0.100
Changes at 6 weeks				
Any change	35%	42%	1.3 (0.7–2.4)	0.356
Exercise	22%	26%	1.2 (0.6–2.3)	0.612
Diet	23%	29%	1.4 (0.7–2.7)	0.323
Supplements	12%	7%	0.5 (0.2–1.5)	0.226
Medications	9%	11%	1.3 (0.5–3.3)	0.640
Other	7%	3%	0.4 (0.1–1.7)	0.217
Changes at 6 months				
Any change	36%	48%	1.6 (0.9–2.9)	0.104
Exercise	23%	35%	1.8 (1.0–3.5)	0.064
Diet	25%	35%	1.6 (0.9–3.1)	0.126
Supplements	10%	7%	0.6 (0.2–1.8)	0.356
Medications	15%	14%	0.9 (0.4–2.0)	0.783
Other	7%	3%	0.5 (0.1–1.9)	0.270

Percentages, and means and 95% confidence intervals of odds ratios were estimated using logistic regression or generalized estimating equations using logit linking functions and binomial distributions.

Within the GS arm, however, differences were observed by risk status (Table 3). The odds that physician-participants recommended a behavior change during disclosure sessions increased by 68% for each high-risk polygenic risk finding (*p* = 0.049). Similarly, the odds that patient-participants reported a health behavior change increased by 49% at 6 weeks and 52% at 6 months for each high-risk polygenic risk finding (*p* = 0.049 and 0.032, respectively).

**Table 3:** Associations between high-risk results, provider recommendations, and patient-reported health behavior changes.

Time point/behavior	Monogenic disease risk (ref: none) OR (95% CI)	*p*	Cardiometabolic risk prediction (per condition in 80th percentile risk) OR (95% CI)	*p*
Recommendations				
Any change	0.59 (0.15–1.84)	0.391	1.68 (1.07–2.72)	0.027
Health behavior	0.79 (0.21–2.50)	0.702	1.41 (0.88–2.32)	0.161
Medication	1.51 (0.19–8.41)	0.652	1.99 (0.94–4.97)	0.097
Changes at 6 weeks				
Any change	1.72 (0.62–4.79)	0.292	1.49 (1.00–2.23)	0.049
Exercise	1.94 (0.66–5.74)	0.228	1.54 (0.99–2.41)	0.057
Diet	1.60 (0.55–4.67)	0.387	1.27 (0.83–1.96)	0.266
Supplements	1.73 (0.30–9.92)	0.534	1.25 (0.54–2.86)	0.602
Medications	0.37 (0.04–3.21)	0.366	1.28 (0.64–2.56)	0.474
Other	NA^a^	0.568	0.48 (0.08–2.94)	0.424
Changes at 6 months				
Any change	2.11 (0.74–6.03)	0.160	1.52 (1.04–2.24)	0.032
Exercise	2.39 (0.85–6.72)	0.098	1.59 (1.05–2.40)	0.029
Diet	1.88 (0.67–5.27)	0.227	1.34 (0.91–1.98)	0.134
Supplements	0.79 (0.08–7.39)	0.832	1.23 (0.59–2.57)	0.571
Medications	0.29 (0.03–2.49)	0.258	1.50 (0.83–2.72)	0.175
Other	2.03 (0.16–25.50)	0.578	1.06 (0.32–3.53)	0.918

High-risk results were defined in two ways: (1) identification of an unexpected monogenic disease risk (analyses exclude two patients who were previously diagnosed with hemochromatosis, who received monogenic disease risk findings on their GS report) and (2) number of polygenic predictions about cardiometabolic traits (range: 0–8) where patients were identified to be in the 80th percentile for genetic risk, per polygenic risk predictions. Means and 95% confidence intervals of odds ratios were estimated using logistic regression or generalized estimating equations using logit linking functions and binomial distributions.

^a^An odds ratio could not be estimated, because no participant with an unexpected monogenic disease risk reported a change to this category at 6 weeks post disclosure. The *p*-value was estimated using a Fisher’s exact test on available data.

### Psychological impact

Twenty-eight percent of patient-participants in the GS arm and 29% of patient-participants in the control arm reported Hospital Anxiety and Depression Scale (HADS) scores indicating at least moderate anxiety or depression at one or more post-disclosure time point, with no differences observed by randomization status (*p* > 0.99). Analyses of general anxiety and depression (Table 4) showed mean scores well below cutoffs for mood disorders, regardless of randomization status, at all time points and data supported equivalence between randomization arms at all time points. Moreover, at 6 weeks post disclosure, mean scores were lower in the GS arm compared to the control arm on measures of depression (difference: −0.7, *p* = 0.015) and positive impact (difference: −2.1, *p* = 0.001), suggesting lower depression and more positive feelings among those receiving GS. Cohort-specific analyses (Supplementary Table 2) were generally consistent with these findings. In the GS arm, differences in positive impact observed at 6 weeks persisted at 6 months among cardiology patient-participants (difference: −1.9, *p* = 0.046), whereas lower depression scores among primary-care patient-participants were observed at post disclosure (difference: −0.8, *p* = 0.046) and at 6 months (difference: −0.8, *p* = 0.037).

**Table 4:** Psychological outcomes on continuous measures.

Time point/outcome	Control (*n* = 101)	GS (*n* = 99)	Difference (95% CI)	*p*
Baseline^a^				
HADS: Anxiety	5.1 (0.2)	5.1 (0.2)		
HADS: Depression	2.3 (0.2)	2.3 (0.2)		
Post-disclosure				
HADS: Anxiety	4.8 (0.3)	4.9 (0.2)	0.1 (−0.6 to 0.9)	0.790
HADS: Depression	2.7 (0.2)	2.1 (0.2)	−0.6 (−1.0 to 0.0)	0.052
6 Weeks				
HADS: Anxiety	4.1 (0.2)	3.6 (0.3)	−0.5 (−1.2 to 0.2)	0.166
HADS: Depression	2.4 (0.2)	1.7 (0.2)	−0.7 (−1.2 to −0.2)	0.015
MICRA: Distress	0.2 (0.1)	0.4 (0.1)	0.2 (−0.1 to 0.6)	0.212
MICRA: Uncertainty	1.2 (0.2)	1.6 (0.3)	0.4 (−0.3 to 1.2)	0.312
MICRA: Positive	10.0 (0.4)	7.9 (0.4)	−2.1 (−3.3 to −0.9)	0.001
6 Months				
HADS: Anxiety	4.7 (0.3)	5.0 (0.2)	0.3 (−0.5 to 1.0)	0.500
HADS: Depression	2.6 (0.2)	2.2 (0.2)	−0.3 (−0.8 to 0.2)	0.244
MICRA: Distress	0.4 (0.1)	0.5 (0.2)	0.1 (−0.3 to 0.6)	0.613
MICRA: Uncertainty	1.4 (0.3)	1.7 (0.3)	0.3 (−0.4 to 1.3)	0.452
MICRA: Positive	10.9 (0.4)	10.1 (0.5)	−0.8 (−2.1 to 0.6)	0.260

Means (SEs) of scores were estimated using generalized estimating equations using linking functions and distributions that varied by outcome.

^a^Baseline scores represent the means and SEs across arms because scores at other time points are adjusted for differences at baseline. Unadjusted means for the control and GS arms at baseline were 4.8 vs. 5.4, respectively, for anxiety (*p* = 0.178) and 2.3 vs. 2.4, respectively, for depression (*p* = 0.661).

Analyses of affective responses (Supplementary Table 3) showed that patient-participants in the GS arm were more likely than patient-participants in the control arm at all post-disclosure time points to report feeling happy, empowered, and relieved by their test results (all *p* < 0.001). They were also less likely to feel disappointed at all time points (all *p* ≤ 0.004), although they were more likely to feel confused (all *p* ≤ 0.013). Immediately post disclosure, patient-participants in the GS arm were more likely than patient-participants in the control arm to report feeling worried about their test results (39% vs. 22%, respectively, *p* = 0.011), although differences did not persist at 6 weeks and beyond. Analyses of affective responses within the primary-care and cardiology cohorts (Supplementary Table 2) were mostly consistent with analyses across cohorts.

Analyses restricted to the GS arm also showed differences on psychological measures according to risk status (Supplementary Table 4). Interestingly, patient-participants who received results about an unexpected monogenic disease risk scored lower than patient-participants who were not identified with an unexpected monogenic disease risk on measures of depression immediately post disclosure (2.3 vs. 3.5, respectively, *p* = 0.008) and anxiety at 6 weeks (3.2 vs. 4.1, respectively, *p* = 0.009). Similarly, patient-participants’ uncertainty scores were lower when patient-participants had more high-risk polygenic risk predictions (−0.5 points per finding, *p* = 0.029).

### Mediation analyses

Scores on five psychological measures were associated with both randomization status and the likelihood of reporting a health behavior change at *p* < 0.05 in bivariate analyses: general depression, positive impact, and happy, empowered, and relieved feelings. Mediation analyses summarized in Table 5 suggested that GS prompted health behavior change by making patient-participants feel a greater positive impact from testing or more happy, empowered, or relieved about the information they received (all *p* < 0.001 for indirect associations between GS and health behavior changes). No indirect effects were observed for physician recommendations for lifestyle change (*p* = 0.195). Mediation analyses only examined the associations between randomization status, psychological outcomes or provider recommendations, and health behavior changes, and omitted variables about high-risk GS results.

**Table 5:** Summary of mediation analyses.

Mediator	Indirect effect	*p*	Direct effect	*p*
HADS: Depression	3% (0% to 6%)	0.065	9% (−4% to 23%)	0.186
MICRA: Positive	7% (3% to 12%)	<0.001	4% (−9% to 18%)	0.559
Emotions				
Happy	8% (1% to 16%)	<0.001	4% (−12% to 19%)	0.640
Empowered	10% (4% to 17%)	<0.001	1% (−13% to 16%)	0.782
Relieved	10% (3% to 17%)	<0.001	2% (−13% to 17%)	0.753

Analyses examined whether the impact of randomization to GS on health behavior changes reported at 6 months were mediated by psychological responses at 6 weeks. Indirect effects are the differences between the GS and control arms in the likelihood of reporting a health behavior change that could be attributed to the impact of GS on potential mediators. Direct effects are the differences between the GS and control arm in the likelihood of reporting a health behavior change after controlling for the potential mediators.

## Discussion

The MedSeq Project is a pilot randomized trial that provides preliminary evidence about the behavioral and psychological impact of GS, to inform future research. Results supported the hypothesis that GS would not increase risks for anxiety or depression and showed that patient-participants who received GS felt more positive about the information in the short-term. Results from exploratory analyses also provided some evidence that polygenic risk predictions for cardiometabolic traits may be able to motivate health behavior changes. Finally, exploratory analyses suggested a greater likelihood of impact of GS on health behaviors if patient-participants had positive psychological responses from receiving their GS results.

Overall, findings present early evidence that is somewhat encouraging about the potential behavioral and psychological impact of integrating GS into patient care. It is notable that physician- and patient-participants were more likely to recommend and report health behavior changes when GS results indicated greater polygenic risks for cardiometabolic traits. This could suggest that physician- and patient-participants were both sensitive to the number of risks identified and to the ability to reduce cardiac risks with lifestyle modifications. Our findings align with two recently published studies showing benefits from disclosing genetic risk information about cardiovascular disease^14,28^. Common to both these studies—and ours—are patient populations that were not recruited based on a preexisting interest in reducing cardiovascular disease risks. It is possible that the behavioral impact of genetic risk disclosure is greatest for individuals when the information is unexpected^18^. Another explanation is that the MedSeq Project attracted patients who were motivated to enact health behavior changes, as was observed in related work^29^. A third possibility is that patients with low-risk genomic findings were falsely reassured by their findings, and that they were less likely to make health behavior changes than comparable patient-participants in the control arm. We did not test this explanation in this manuscript, but it may explain the null behavioral findings in comparisons of randomization arms despite a greater likelihood of behavior changes with increased risks for cardiometabolic traits.

Findings from our study may also help allay fears about the distress that GS may cause, particularly regarding secondary findings^6^. As noted in our prior reported meta-analyses of post-disclosure outcomes in patient-participants randomized to GS^21^, we found that patient-participants in our study coped well with GS findings, even when results showed a high and unanticipated risk for genetic disorders. In fact, in the analyses presented here, patient-participants who were identified with unexpected monogenic disease risks had lower depression scores immediately post disclosure and lower anxiety scores at 6 weeks post disclosure than patient-participants who did not have unexpected monogenic disease risks. One explanation for these counter-intuitive patterns is that providers may have been more engaged with these patients, while explaining findings on the reports. Moreover, these patients may have felt a sense of relief. Patients who had unexpected monogenic disease risks were either asymptomatic for the conditions and unlikely to develop them in the future or they were already symptomatic and genomic findings helped to explain their conditions^20,25^.

Psychological outcomes were particularly interesting given that the breadth of information provided in the MedSeq Project was far greater than typical GS protocols, where secondary findings may be limited to conditions where proven prevention and treatment options exist^30,31^. Our study disclosed pathogenic, likely pathogenic, and variants of unknown significance where evidence favored pathogenicity in over 4600 genes for monogenic conditions, as well as carrier status, pharmacogenomic information, and polygenic risk predictions for cardiometabolic traits. The large amount and different types of information in our GS reports likely explains why approximately one in six MedSeq patient-participants also reported some confusion at 6 months follow-up and prior reported results showed that patient-participants who received GS reported poorer understandings of the information they received^32^. It is possible that positive responses to GS may have been even stronger if findings were easier to understand or strategies were more explicitly discussed^33^. On the other hand, uncertainty scores at 6 months were lower among patients with more polygenic risks for cardiometabolic traits. It is possible that uncertainty scores were lower, because physicians were more engaged with these patients, as suggested in prior reported MedSeq analyses^34^.

Another important finding from our analyses was the role of positive psychological responses in predicting behavioral outcomes. Prior work has speculated about how psychological responses to genomic test results might lead to behavior change^18,19^, but our study is one of the first to examine this relationship empirically. Interestingly, although many health behavior theories posit that individuals will make lifestyle adjustments as an adaptive response to negative emotions such as fear and anxiety^35–37^, our data suggest that GS may have motivated health behavior changes among those with positive emotional responses to receiving their GS results. Positive emotional states may facilitate health behavior change by enhancing resolve and making individuals more resilient to barriers such as tiredness and unaccommodating built environments^38,39^. These findings add weight to calls for better understanding of psychological responses to genomic information, including positive emotions^40^. Notably, positive emotional outcomes are often omitted from research on genomic testing^15,41,42^.

Limitations to this study include limited sample size and statistical power in exploratory analyses, which did not account for multiple hypothesis testing or potential confounding with provider. Analyses did not allow us to determine what type of FH or genomic information motivated self-reported behavior changes. Provider- and patient-participants were early adopters and thus were self-selected, highly educated and affluent, and motivated to receive information from GS. Physician understandings of FH or genomic results were not assessed, although analyses showed that primary-care physicians responded to unexpected monogenic disease risks appropriately, in general^43^. Patients were primarily non-Hispanic White and were recruited by a trusted physician into a study run by a well-respected research institution. All patients in the cardiology cohort received genetic testing for their condition previously, including some who received exome or GS. Findings may not generalize to other study populations or contexts, including those where improving lifestyles is the primary patient outcome. In those contexts, more appropriate comparators would be interventions with more proven efficacy.

Data analytic issues were also limitations. We did not track resources that physician-participants used to respond to the reports they received, including clinical decision support sheets for FH. Some of the survey measures were not validated and were dichotomized in analyses that grouped response options such as “slightly” and “extremely” together. Health behavior measures were simple yes/no questions about broad domains, have not been validated, did not provide insight about whether changes were clinically meaningful, and may have been subject to self-report bias. Mediation analyses were limited to testing whether 6-week psychological outcomes predicted 6-month behavioral outcomes, as posited prior to the trial^18,19^. The reverse relationship and reciprocal relationship also merit examination^38,44^. The definitions of “high-risk results” were also created for this study, with an emphasis on unexpected genomic information. Given that we did not define high-risk FH, a comparable definition of high-risk results were not incorporated for the control arm. Alternative approaches to characterizing “high-risk results” could include allowing patient-participants to decide for themselves whether they received high-risk results or developing consensus approaches investigators from other studies of genomic sequencing. Analyses of polygenic risk predictions may also benefit from creating a measure of low-risk results, given the potential for false reassurance^45^. Such analyses could implement a measure with an emphasis on the number of conditions where patients were at the lowest percentiles of genetic risk for cardiometabolic traits.

Nevertheless, findings from our pilot trial present critical early evidence about the psychological and health behavior impact of integrating GS into broad clinical care, to inform future research with more diverse participants. As the accessibility of GS expands, capitalizing on GS’s ability to identify unknown health risks may have health promotion benefits.

## Methods

### Study design and participants

The MedSeq Project was a pilot randomized trial to understand the impact of integrating GS into two clinical contexts: cardiology care of patients with diagnoses of genetic conditions and primary care of healthy patients. These contexts were targeted to provide insight about two archetypal scenarios, about how GS could be integrated into clinical care, as follows: (i) disease-specific genomic medicine, where testing focuses on identifying variants in relevant disease-associated genes; and (ii) general genomic medicine, where testing informs routine preventive medicine^22^. Details of the study design, genomic analyses, recruitment, and enrollment have been published elsewhere^22,29,46–48^. Briefly, 9 cardiologists and 11 primary-care physicians were enrolled into the study and enlisted their patients into the study protocol. Study staff recruited patients using letters and via telephone. Eligible primary-care patients were generally healthy adults aged 40–65 years when approached for recruitment. Eligible cardiology patients were adults of any age with diagnoses of HCM/DCM, who had previously received prior or concurrent panel-based genetic testing, to try to find a genetic explanation for their condition. Exclusion criteria included ongoing pregnancies and lack of English fluency. All patients with diabetes and primary-care patients with cardiovascular disease were also excluded because GS reports included polygenic risk predictions for associated phenotypes^48^. Patients completed an FH tool, provided blood for potential sequencing, and completed a baseline survey.

Patients who completed the baseline survey were randomized to review their FH alone (control arm) or in conjunction with a GS report (GS arm) during a disclosure meeting with their physician. Participants’ randomization statuses were computer-assigned and drawn in sequence from sealed envelopes after participants completed the baseline questionnaire. Cardiology patients in both arms also reviewed prior HCM/DCM genetic testing results. FH and GS reports, if applicable, were sent to patients’ physician-participants ~1 week in advance of results disclosure sessions.

Methods for GS analysis, interpretation, results presentation, and sample GS reports were published previously^29,49^. FH reports were generated using a customized version of the U.S. Surgeon General’s “My Family Health Portrait” web tool^50^ and included pedigrees and written summaries. FH reports did not highlight potentially concerning results, but were accompanied by six clinical decision support modules to help physicians interpret and manage FH information about breast and colon cancer, coronary artery disease, type 2 diabetes, glaucoma, and osteoporosis^22^. GS reports communicated monogenic disease risks, regardless of whether the associated conditions had proven prevention strategies. GS reports also included carrier status for autosomal recessive conditions, pharmacogenomic information about five drugs, predicted lipid profiles, blood antigen predictions, and polygenic risk information about eight cardiometabolic traits as follows: abdominal aortic aneurysm, atrial fibrillation, coronary heart disease, type 2 diabetes, hypertension, obesity, platelet aggregation, and QT prolongation. At the time of the study, the laboratory examined between 3 and 70 loci for each trait to generate polygenic risk estimates^48^. FH and GS reports did not include numeric estimates of disease incidence, although polygenic risk predictions included estimates of relative risks and percentiles of genetic risk^51^.

Physician-participants were responsible for communicating FH and GS reports, as applicable, to their patients at their own discretion. Reports were typically sent by MedSeq Project staff to physician-participants a week in advance of a disclosure visit. At any time, providers could contact a Genome Resource Center staffed by study-affiliated medical geneticists and genetic counselors with questions. The aforementioned decision support modules were also provided to help with managing FH reports. No other clinical decision support was provided. FH and GS reports were integrated into patients’ medical records after results were disclosed to patient-participants.

Patient-participants were surveyed immediately after disclosure and again 6 weeks and 6 months post disclosure. Physician-participants completed brief surveys immediately after disclosure where they reported any recommendations they made in response to FH or GS reports regarding medications and health behaviors. Patients provided written informed consent at their baseline visit, whereas physicians provided written informed consent during an in-person educational session prior to returning any results to patients. Participants were followed from 2012 to 2016. The Mass General Brigham (formerly Partners HealthCare) Human Research Committee and the Baylor College of Medicine Institutional Review Board both approved this study. Written informed consent was obtained in-person from participants. Our study conforms to the Declaration of Helsinki. The study was registered at ClinicalTrials.gov with identifier NCT01736566 on 29 November 2012.

### Measures

Patients reported demographic information on the baseline survey. Exploratory behavioral and psychological outcomes of interest are described below. Psychological measures in the MedSeq Project were selected to facilitate analyses with other studies that were also part of the CSER Consortium^19^. Behavioral measures were broad in scope, because specific changes to lifestyle were not a focus of our study, nor was GS tailored to address specific conditions that may benefit from targeted prevention. Given the pilot nature of this study, most measures were implemented to provide insight that could inform future trials rather than to make decisive conclusions about behavioral and psychological outcomes.

### Health behaviors

#### Physician recommendations

Immediately post disclosure, physician-participants completed checklists that included novel items asking yes/no questions about whether physician-participants did “change a medication” or “recommend a health behavior change(s)”, and also asked what information from the FH or GS reports motivated the discussion.

#### Changes to health behaviors

Patient surveys administered 6 weeks and 6 months post disclosure included items adapted from multiple trials of genetic risk for Alzheimer’s disease^14,51–53^ that asked, “Have you made any of the following health or wellness changes that were specifically motivated by the information you discussed with your doctor?” Response options were “diet”, “exercise”, “use of vitamins/herbal supplements”, “use of medications”, and “other”. An aggregated measure of “any change” was created to indicate whether patients endorsed a change to any of the five response options.

### Psychological outcomes

#### General anxiety and depression

At each time point, including baseline, patients completed the Hospital Anxiety and Depression Scale, a commonly used 14-item instrument that addresses general anxiety and depression, and has been validated in general adult populations^43,54^. Scores on subscales assessing anxiety and depression range from 0 to 21 (normal: 0–7; mild: 8–10; moderate: 11–14; severe: 15–21).

#### Test-related impact

As part of the 6-week and 6-month follow-up questionnaires, patients completed an adapted version of the Multidimensional Impact of Cancer Risk Assessment (MICRA)^55^. The scale assessed patients’ psychological responses to the information they received during MedSeq on three subscales as follows: distress, uncertainty, and positive impact. Patients rated how much “the information you discussed with your doctor as part of this study” made them experience feelings such as upset, happy, and guilty during the prior week. Response options of “not at all”, “a little”, “somewhat”, “a good deal”, and “a great deal” were scored 0–4, respectively, and summed into the subscales measuring distress (6 items, range 0–24, Cronbach’s *α* = 0.86), uncertainty (9 items, range 0–36, *α* = 0.77), and positive impact (4 reverse-scored items, range 0–16, *α* = 0.75). Higher scores on the distress and uncertainty subscales indicated stronger distress and uncertainty, whereas higher scores on the positive impact subscale indicated stronger negative feelings. Although the MICRA was originally developed and validated to assess responses to genetic susceptibility to hereditary breast and ovarian cancer, it has been adapted and undergone preliminary validation work that included asymptomatic adults to assess the impact of genetic risk disclosure for other contexts, including genetic susceptibility testing for Alzheimer disease^56^ and genomic sequencing^57^. Of note, the MICRA has been adapted in multiple research consortia to examine the test-related impact of genomic sequencing^19,58^.

#### Affective responses

Novel items administered in each follow-up survey assessed whether the information patients discussed with their doctor made them feel happy, empowered, relieved, worried, confused, or disappointed since completing their last survey. Response options included “not at all”, “slightly”, “moderately”, “very”, and “extremely”. These items were also administered at baseline and patients were asked to rate the likelihood (“no”, “probably no”, “probably yes”, and “yes”) that their results would make them feel these emotions.

### Data analysis

Given the exploratory nature of this pilot trial, we did not set enrollment targets to power specific analyses and instead set targets for disclosure sessions with 100 primary-care and 100 cardiology patients. However, we tested the following a priori hypotheses: (a) patient-participants in the GS arm would be more likely than patient-participants in the control arm to report a change to at least one health behavior and (b) distress would be equivalent between patient-participants in the GS and control arms. Analyses included all patient-participants who had disclosure sessions where FH results and GS results, if applicable, were discussed. Analyses omitted four randomized patient-participants who did not have disclosure sessions, including two who died, one who withdrew due to concerns about potential genetic discrimination, and one who was lost to follow-up. Changes to smoking status were assessed but omitted from reporting, because only four smokers enrolled in the study. Unless otherwise noted, we dichotomized single-item measures of affective responses to compare responses of “slightly”, “moderately”, “very”, and “extremely” against “not at all”, given ordinal response options and highly skewed data.

Analyses comparing randomization arms on physician-participant recommendations used logistic regression and controlled for cardiology or primary-care cohort. We used generalized linear models fit with generalized estimating equations in longitudinal analyses for all other outcomes, incorporating an independent working correlation structure with robust standard errors to account for repeated measures within a participant. For anxiety, depression, distress, and uncertainty, separate models for each outcome used a log link and γ-distribution to compare scores by randomization status given skewed data. A value of 1 was added to these measures to shift the distribution away from zero. For models that analyzed positive impact, we used an identity link and normal distribution to compare scores by randomization status. For models that examined dichotomous outcomes (health behavior changes and affective responses), we used a logit link and binomial distribution. Primary models included terms for randomization status, time as a categorical variable, time-randomization interaction, and baseline score, where applicable. We also ran models that adjusted for whether prior genetic testing had identified a variant associated with a cardiology patient’s DCM or HCM diagnosis, but omitted this variable from all final models, because it was not associated with outcomes of interest (all *p* > 0.05). Cohort-specific analyses were conducted with models that also included terms for cohort, time–cohort interaction, randomization–cohort interaction, and time–cohort–randomization interaction.

In the GS arm, we conducted exploratory analyses to examine the impact of high-risk genetic findings, defined as (A) the presence of an unexpected monogenic disease risk or (B) polygenic risk predictions for cardiometabolic traits in the 80th percentile or higher (i.e., higher risk predictions than 80% of the population). Monogenic findings associated with HCM or DCM for cardiology patients were omitted from these analyses given that these indication-based findings had all been disclosed previously, with only one exception^25^. Analyses of the impact of disclosing unexpected monogenic disease risks were conducted by including a term in regression models that indicated the presence or absence of monogenic disease risk. Analyses of the impact of disclosing polygenic risk predictions were conducted by including a term in regression models that indicated the number of high-risk polygenic risk predictions (0–8) a patient was found to have.

Finally, exploratory analyses examined whether indirect relationships existed between randomization to GS and health behavior changes, where GS may have caused a change to a psychological outcome, which was itself associated with behavior changes. These analyses treated ordinal affective response variables as continuous variables given the size of the study and number of response options^59^. Psychological outcomes were considered for mediation if their scores at 6 weeks had bivariate associations at *p* < 0.05 with randomization status and bivariate associations at *p* < 0.05 with health behavior change at 6 months. We also tested whether physician recommendations any mediation effects of physician recommendations, given the importance of providers’ responses on patient outcomes. We conducted model-based causal mediation analyses using the *mediate* package in R, to estimate the direct and indirect effects of randomization to GS^60^. First, we created regression models to predict scores at 6 weeks of potential mediators (i.e., selected psychological outcomes and provider recommendations) based on randomization status. Second, we created probit regression models to predict the likelihood that patient-participants would report a health behavior change at 6 months based on randomization status, after controlling for scores of potential mediators at 6 weeks. Mediation analyses with 1000 simulations used parameter estimates from these models to estimate indirect effects (i.e., the differences between randomization arms that could be attributed to the impact of GS on the mediator) and direct effects (i.e., the differences between randomization arms that were independent of the impact of GS on the mediator). Confidence intervals were estimated based on nonparametric bootstrapping.

As this was a pilot trial, we asserted statistical significance at *α* = 0.05 on all outcomes and used two-sided tests in all analyses. To test the hypothesis that patient-participants randomized to the GS arm would show equivalent levels of anxiety and depression as patient-participants randomized to the control arm, we asserted equivalence if confidence intervals for the mean differences on HADS subscales were <1.5 points, as defined as the minimal clinically important difference in other studies^61^. Per best practice recommendations, missing data were handled with multiple imputation^62,63^. We used the *mice* package for R and ran 100 iterations to create each of 20 imputed datasets using fully conditional specification^64^. We also ran statistical models on available data and highlight instances where available-case analyses did not support analyses of imputed data.

### Reporting summary

Further information on research design is available in the Nature Research Reporting Summary linked to this article.

## Supplementary information

Supplementary Information

Reporting Summary

## Data Availability

The datasets generated and/or analyzed during the current study are not publicly available to protect the privacy of participants, but are available from the corresponding author on reasonable request.

## Code availability

Analytic code for these analyses was written for R version 4.0.3, including the *mice* package version 3.8.0. This code is available from the corresponding author on reasonable request.
