# Supplementary material for: Behavioral and psychological impact of genome sequencing: a pilot randomized trial of primary care and cardiology patients
Source: NPJ Genom Med. 2021 Aug 24;6:72. doi: 10.1038/s41525-021-00236-2 (PMC8384838; doi:10.1038/s41525-021-00236-2)
Supplement: Supplementary file 1 — Supplementary Information [file 41525_2021_236_MOESM1_ESM.pdf]

Supplementary File Contents

Supplementary Figure 1: Participant flow. 2

Supplementary Table 1: Health behavior changes following results disclosure, by cohort 3

Supplementary Table 2. Mean scores and standard errors on psychological outcomes, by cohort 4

Supplementary Table 3. Affective responses, by randomization arm and time point 6

Supplementary Table 4. Associations between high-risk results and psychological outcomes 7

Supplementary Note 1: Additional Members of the MedSeq Project Team 9

Supplementary Figure 1: Participant flow

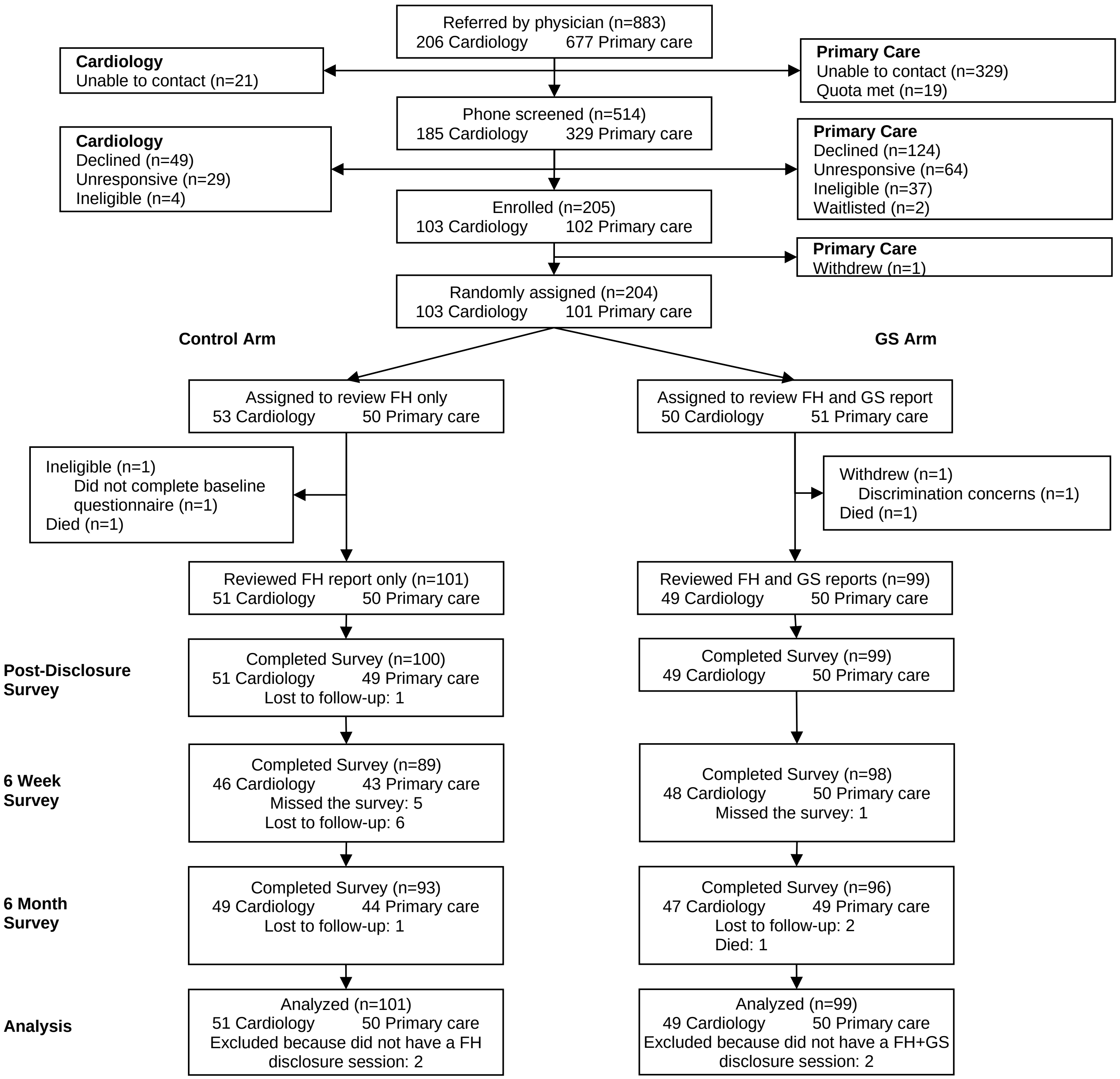

Supplementary Table 1: Health behavior changes following results disclosure, by cohort

	Cardiology				Primary Care			
	**Control (n=51)**	**GS (n=49)**	**OR (95% CI)**	**p**	**Control (n=50)**	**GS (n=50)**	**OR (95% CI)**	**p**
Provider Recommendation for Change at Disclosure								
Any change	4%	14%	4.1 (0.8 to 20.7)	0.090	20%	32%	1.9 (0.8 to 4.7)	0.174
Health Behavior	4%	14%	4.1 (0.8 to 20.7)	0.090	20%	24%	1.3 (0.5 to 3.3)	0.630
Medication	0%	0%	1.0 (NA)	>0.999	4%	14%	3.9 (0.8 to 19.8)	0.100
Patient-Reported Changes at 6 Weeks								
Any change	33%	35%	1.1 (0.5 to 2.6)	0.816	37%	48%	1.5 (0.7 to 3.5)	0.298
Exercise	19%	23%	1.3 (0.5 to 3.5)	0.601	26%	28%	1.1 (0.4 to 2.8)	0.840
Diet	22%	32%	1.7 (0.7 to 4.3)	0.264	24%	26%	1.1 (0.4 to 3.0)	0.775
Supplements	7%	8%	1.2 (0.3 to 5.9)	0.797	18%	6%	0.3 (0.1 to 1.2)	0.088
Medications	12%	11%	0.9 (0.2 to 3.5)	0.906	7%	12%	1.9 (0.4 to 8.3)	0.413
Other	7%	2%	0.3 (0.0 to 3.0)	0.306	8%	4%	0.5 (0.1 to 3.3)	0.475
Patient-Reported Changes at 6 Months								
Any change	42%	55%	1.7 (0.8 to 3.9)	0.182	31%	41%	1.6 (0.7 to 3.7)	0.311
Exercise	29%	44%	1.9 (0.8 to 4.5)	0.120	17%	27%	1.8 (0.6 to 5.0)	0.266
Diet	28%	38%	1.6 (0.7 to 3.7)	0.308	22%	33%	1.8 (0.7 to 4.6)	0.237
Supplements	10%	9%	0.8 (0.2 to 3.3)	0.808	11%	4%	0.4 (0.1 to 2.3)	0.299
Medications	21%	15%	0.7 (0.2 to 2.0)	0.483	9%	12%	1.4 (0.4 to 5.2)	0.640
Other	6%	4%	0.7 (0.1 to 4.2)	0.662	8%	2%	0.3 (0.0 to 2.8)	0.271

Supplementary Table 2. Mean scores and standard errors on psychological outcomes, by cohort
Scores were estimated using generalized estimating equations using linking functions and distributions that varied by outcome.

	Cardiology				Primary Care			
	Control (n=51)	GS (n=49)	Difference (95% CI)	p	Control (n=50)	GS (n=50)	Difference (95% CI)	p
Baseline[1]								
HADS: Anxiety	5.1 (0.3)				5.0 (0.3)			
HADS: Depression	2.9 (0.2)				1.8 (0.2)			
Immediately post-disclosure								
HADS: Anxiety	4.7 (0.4)	4.4 (0.3)	-0.3 (-1.2 to 0.8)	0.548	4.8 (0.4)	5.4 (0.3)	0.5 (-0.5 to 1.8)	0.352
HADS: Depression	2.8 (0.3)	2.6 (0.2)	-0.2 (-0.9 to 0.6)	0.552	2.5 (0.3)	1.7 (0.3)	-0.8 (-1.5 to 0.0)	0.046
6 weeks								
HADS: Anxiety	4.1 (0.3)	3.4 (0.3)	-0.7 (-1.5 to 0.4)	0.195	4.2 (0.3)	3.9 (0.4)	-0.4 (-1.3 to 0.8)	0.492
HADS: Depression	2.6 (0.3)	2.0 (0.2)	-0.6 (-1.1 to 0.1)	0.080	2.3 (0.4)	1.5 (0.2)	-0.8 (-1.5 to 0.1)	0.064
MICRA: Distress	0.2 (0.2)	0.6 (0.2)	0.4 (-0.1 to 1.1)	0.126	0.2 (0.1)	0.2 (0.1)	0.0 (-0.3 to 0.3)	0.916
MICRA: Uncertainty	1.6 (0.3)	2.3 (0.5)	0.8 (-0.3 to 2.2)	0.163	0.8 (0.3)	0.8 (0.3)	0.0 (-0.7 to 1.0)	0.966
MICRA: Positive	9.4 (0.6)	7.0 (0.5)	-2.4 (-4.0 to -0.8)	0.004	10.7 (0.5)	8.8 (0.7)	-1.9 (-3.6 to -0.2)	0.032
6 months								
HADS: Anxiety	4.5 (0.3)	4.8 (0.3)	0.2 (-0.8 to 1.4)	0.674	4.9 (0.4)	5.1 (0.3)	0.3 (-0.7 to 1.4)	0.606
HADS: Depression	2.7 (0.3)	3.0 (0.2)	0.3 (-0.4 to 1.1)	0.473	2.4 (0.3)	1.6 (0.3)	-0.8 (-1.4 to -0.1)	0.037[2]
MICRA: Distress	0.4 (0.2)	0.6 (0.3)	0.2 (-0.3 to 1.1)	0.501	0.3 (0.1)	0.4 (0.3)	0.0 (-0.5 to 0.8)	0.967
MICRA: Uncertainty	2.0 (0.5)	2.4 (0.5)	0.4 (-0.8 to 2.1)	0.573	0.7 (0.2)	1.0 (0.3)	0.2 (-0.4 to 1.2)	0.507
MICRA: Positive	10.8 (0.6)	9.0 (0.7)	-1.9 (-3.7 to 0.0)	0.046	11.0 (0.7)	11.3 (0.7)	0.3 (-1.6 to 2.2)	0.757

[1] Baseline scores represent the means and standard errors across arms because scores at other time points are adjusted for differences at baseline. For the cardiology cohort, unadjusted means for the control and GS arms at baseline were 4.5 vs 5.7, respectively, for anxiety (p=0.065) and 2.7 vs 3.0, respectively, for depression (p=0.606). Within the primary care cohort, unadjusted means for the control and GS arms at baseline were 5.0 vs 5.1, respectively, for anxiety (p=0.0.893) and 1.8 vs 1.8 for depression (p=0.889).
[2] Difference was non-significant (p=0.070) in available case analyses.

Supplementary Table 2. Psychological outcomes, by cohort (continued).

	Cardiology				Primary Care			
	Control (n=51)	GS (n=49)	Difference (95% CI)	p	Control (n=50)	GS (n=50)	Difference (95% CI)	p
Immediately post-disclosure[1]								
Worried	12%	41%	4.9 (1.7 to 14.0)	0.003	31%	37%	1.3 (0.6 to 3.0)	0.552
Confused	4%	17%	5.1 (1.0 to 27.6)	0.057	4%	18%	5.3 (1.1 to 26.1)	0.041[2]
Disappointed	27%	15%	0.4 (0.2 to 1.2)	0.122	39%	14%	0.3 (0.1 to 0.7)	0.010
Happy	46%	76%	4.1 (1.6 to 10.4)	0.003	58%	90%	6.5 (2.2 to 19.5)	<0.001
Empowered	45%	76%	3.7 (1.5 to 9.2)	0.005	44%	80%	5.2 (2.1 to 13.2)	<0.001
Relieved	41%	71%	3.5 (1.5 to 8.2)	0.004	40%	84%	7.6 (2.9 to 19.8)	<0.001
6 Weeks[1]								
Worried	18%	21%	1.2 (0.4 to 3.3)	0.741	21%	26%	1.3 (0.5 to 3.5)	0.547
Confused	3%	19%	8.8 (1.2 to 67.3)	0.036	4%	18%	5.7 (0.8 to 40.9)	0.084
Disappointed	41%	15%	0.3 (0.1 to 0.7)	0.007	43%	4%	0.1 (0.0 to 0.3)	<0.001
Happy	33%	79%	9.0 (3.3 to 24.4)	<0.001	41%	77%	4.7 (1.9 to 11.5)	<0.001
Empowered	36%	78%	6.4 (2.6 to 15.7)	<0.001	38%	66%	3.1 (1.3 to 7.2)	0.008
Relieved	25%	75%	9.3 (3.5 to 24.3)	<0.001	35%	82%	8.5 (3.3 to 21.9)	<0.001
6 Months[1]								
Worried	13%	21%	1.7 (0.6 to 5.0)	0.344	19%	13%	0.6 (0.2 to 2.0)	0.435
Confused[3]	5%	9%	2.0 (0.3 to 11.1)	0.448	0%	24%	NA	<0.001
Disappointed	27%	5%	0.1 (0.0 to 0.6)	0.012	28%	11%	0.3 (0.1 to 1.0)	0.054
Happy	43%	77%	5.0 (1.9 to 13.1)	0.001	44%	81%	5.5 (2.2 to 14.0)	<0.001
Empowered	45%	70%	2.8 (1.2 to 6.6)	0.017	41%	81%	6.2 (2.4 to 16.2)	<0.001
Relieved	31%	72%	5.7 (2.3 to 14.1)	<0.001	31%	82%	10.1 (3.9 to 26.3)	<0.001

[1] Percentages represent the likelihood that a participant rated feeling these feelings about their study information at least "slightly."

[2] Analyses were non-significant (p=0.093) in available case analyses

[3] The statistical model used to estimate an odds ratio for confusion among cardiology patients at 6 months included data from only the 6-month time point. A model that included other time points in interaction with cohort and randomization status, as used for other analyses reported in this table, was unstable because no primary care patients in the control arm reported confusion about their results at 6 months. The p-value for randomization arm comparisons among primary care patients was estimated from available data using Fisher's exact test.

Supplementary Table 3. Affective responses, by randomization arm and time point
Percentages represent the proportion of participants reporting each affective response at least “slightly.” Scores were estimated using generalized estimating equations using logit linking functions and binomial distributions.

	Control (n=51)	GS (n=49)	Difference (95% CI)	p
Immediately Post-disclosure				
Worried	22%	39%	2.3 (1.2 to 4.3)	0.011
Confused	4%	17%	4.8 (1.5 to 15.2)	0.007
Disappointed	33%	15%	0.3 (0.2 to 0.7)	0.004
Happy	52%	83%	4.5 (2.3 to 8.8)	<0.001
Empowered	44%	78%	4.4 (2.3 to 8.3)	<0.001
Relieved	41%	77%	5.0 (2.7 to 9.3)	<0.001
6 Weeks				
Worried	20%	23%	1.3 (0.6 to 2.5)	0.500
Confused	4%	19%	6.3 (1.5 to 25.2)	0.011
Disappointed	42%	10%	0.1 (0.1 to 0.3)	<0.001
Happy	37%	78%	6.1 (3.2 to 11.6)	<0.001
Empowered	37%	72%	4.3 (2.4 to 8.0)	<0.001
Relieved	30%	79%	8.7 (4.5 to 16.6)	<0.001
6 Months				
Worried	16%	17%	1.0 (0.5 to 2.3)	0.919
Confused	3%	16%	6.2 (1.5 to 26.4)	0.013
Disappointed	27%	8%	0.2 (0.1 to 0.6)	0.002
Happy	43%	79%	5.0 (2.6 to 9.4)	<0.001
Empowered	43%	76%	4.1 (2.2 to 7.6)	<0.001
Relieved	31%	77%	7.4 (3.9 to 14.1)	<0.001

Supplementary Table 4. Associations between high-risk results and psychological outcomes
High-risk results were defined in two ways: 1) identification of an unexpected monogenic disease risk, and 2) number of cardiometabolic traits (range: 0-8) where patients were identified to be in the 80th percentile or higher for genetic risk, per polygenic risk predictions.

	Difference in score when an unexpected monogenic risk is disclosed (95% CI)	p	Difference in score per high-risk finding for polygenic risk (95% CI)	p
Immediately Post-Disclosure				
HADS: Anxiety	-0.9 (-1.9 to 0.3)	0.123	-0.1 (-0.6 to 0.4)	0.722
HADS: Depression	-1.2 (-1.9 to -0.4)	0.008	0.2 (-0.3 to 0.7)	0.566
6 Weeks				
HADS: Anxiety	-1.6 (-2.5 to -0.5)	0.009	-0.2 (-0.7 to 0.4)	0.504
HADS: Depression	-0.6 (-1.4 to 0.4)	0.234	-0.2 (-0.6 to 0.3)	0.449
MICRA: Distress	-0.3 (-0.6 to 0.0)	0.076	-0.1 (-0.3 to 0.0)	0.112
MICRA: Uncertainty	-0.6 (-1.4 to 0.6)	0.252	-0.4 (-0.8 to 0.1)	0.146
MICRA: Positive	-0.6 (-3.0 to 1.8)	0.625	0.2 (-0.6 to 1.0)	0.611
6 Months				
HADS: Anxiety	-1.0 (-2.0 to 0.2)	0.100	0.0 (-0.5 to 0.6)	0.911
HADS: Depression	-1.0 (-1.9 to 0.3)	0.125	-0.2 (-0.7 to 0.3)	0.424
MICRA: Distress	0.9 (-0.2 to 3.2)	0.158	-0.1 (-0.2 to 0.1)	0.453
MICRA: Uncertainty	-0.1 (-1.1 to 1.6)	0.928	-0.5 (-0.9 to -0.1)	0.029
MICRA: Positive	0.5 (-2.2 to 3.1)	0.730	0.6 (-0.3 to 1.4)	0.195

Supplementary Table 4 (continued). Associations between high-risk results and psychological outcomes within the GS arm.

	Odds ratio for response when an unexpected monogenic risk is disclosed, compared to no monogenic risk disclosed (95% CI)	p	Change in odds ratio for response per high-risk finding for polygenic risk (95% CI)	p
Immediately Post-Disclosure				
Worried	1.5 (0.6 to 4.3)	0.407	1.2 (0.8 to 1.8)	0.293
Confused	0.2 (0.0 to 1.8)	0.152	1.4 (0.8 to 2.3)	0.234
Disappointed	1.3 (0.3 to 5.5)	0.709	1.2 (0.7 to 2.2)	0.473
Happy	0.5 (0.1 to 1.6)	0.236	1.1 (0.7 to 1.8)	0.605
Empowered	0.8 (0.2 to 2.5)	0.671	0.8 (0.5 to 1.2)	0.257
Relieved	1.1 (0.3 to 3.8)	0.865	1.0 (0.6 to 1.6)	0.999
6 Weeks				
Worried	0.9 (0.2 to 2.9)	0.795	1.2 (0.8 to 1.8)	0.478
Confused	0.4 (0.1 to 2.2)	0.311	0.8 (0.5 to 1.2)	0.250
Disappointed	0.5 (0.1 to 4.6)	0.557	0.9 (0.4 to 2.0)	0.847
Happy	0.7 (0.2 to 2.3)	0.565	1.2 (0.8 to 1.9)	0.401
Empowered	1.1 (0.3 to 3.5)	0.871	0.9 (0.6 to 1.4)	0.554
Relieved	0.7 (0.2 to 2.3)	0.549	1.0 (0.6 to 1.5)	0.989
6 Months				
Worried	1.9 (0.5 to 6.9)	0.310	1.2 (0.8 to 1.9)	0.376
Confused	1.3 (0.3 to 4.9)	0.718	1.1 (0.7 to 1.8)	0.671
Disappointed	0.7 (0.1 to 6.1)	0.730	0.9 (0.4 to 2.2)	0.856
Happy	1.0 (0.3 to 3.4)	0.952	1.1 (0.7 to 1.9)	0.686
Empowered	0.7 (0.2 to 2.2)	0.544	1.0 (0.6 to 1.5)	0.988
Relieved	0.8 (0.3 to 2.6)	0.734	0.9 (0.6 to 1.3)	0.480

Supplementary Note 1: Additional Members of the MedSeq Project Team

Members of the MedSeq Project are as follows: David W. Bates, MD, Wendi N. Betting, Allison L. Cirino, MS, Carolyn Y. Ho, MD, Joel B. Krier, MD, William J. Lane, MD, PhD, Lisa S. Lehmann, MD, PhD, MSc, Calum A. MacRae, MD, PhD, Cynthia C. Morton, PhD, Christine E. Seidman, MD, Shamil R. Sunyaev, PhD, Jason L. Vassy, MD, MPH, Tiffany Nguyen, Eleanor Steffens, Carrie L. B. Zawatsky, MS, CGC, Brigham and Women's Hospital and Harvard Medical School; Christine Y. Lu, MSc, PhD, Harvard Pilgrim Health Care Institute; Samuel J. Aronson, ALM, MA, Ozge Ceyhan-Birsoy, PhD, Matthew S. Lebo, PhD, Kalotina Machini, PhD, Heather M. McLaughlin, PhD, Heidi L. Rehm, PhD, Ellen A. Tsai, PhD, Partners Healthcare Personalized Medicine; Jennifer Blumenthal-Barby, PhD, Hayley Peoples, Baylor College of Medicine, Center for Medical Ethics and Health Policy; Pamela M. Diamond, PhD, University of Texas Houston School of Public Health; Kelly Davis, Peter A. Ubel, MD, Duke University; Peter Kraft, PhD, Harvard School of Public Health; J. Scott Roberts, PhD, University of Michigan; Judy E. Garber, MD, MPH, Dana-Farber Cancer Institute; Tina Hambuch, PhD, Illumina, Inc.; Michael F. Murray, MD, Geisinger Health System; Isaac Kohane, MD, PhD, Sek Won Kong, MD, Boston Children's Hospital; Dmitry Dukhovny, MD, MPH, Oregon Health and Science University.
