# Supplementary material for: Behavioral and psychological impact of genome sequencing: a pilot randomized trial of primary care and cardiology patients
Source: NPJ Genom Med. 2021 Aug 24;6:72. doi: 10.1038/s41525-021-00236-2 (PMC8384838; doi:10.1038/s41525-021-00236-2)
Supplement: Supplementary file 2 — Reporting Summary [file 41525_2021_236_MOESM2_ESM.pdf]

Corresponding author(s): Kurt Christensen

Last updated by author(s): Jun 25, 2021

Reporting Summary

Nature Portfolio wishes to improve the reproducibility of the work that we publish. This form provides structure for consistency and transparency in reporting. For further information on Nature Portfolio policies, see our Editorial Policies and the Editorial Policy Checklist.

Statistics

For all statistical analyses, confirm that the following items are present in the figure legend, table legend, main text, or Methods section.

n/a	Confirmed	
☐	☒	The exact sample size (*n*) for each experimental group/condition, given as a discrete number and unit of measurement
☐	☒	A statement on whether measurements were taken from distinct samples or whether the same sample was measured repeatedly
☐	☒	The statistical test(s) used AND whether they are one- or two-sided<br>*Only common tests should be described solely by name; describe more complex techniques in the Methods section.*
☐	☒	A description of all covariates tested
☐	☒	A description of any assumptions or corrections, such as tests of normality and adjustment for multiple comparisons
☐	☒	A full description of the statistical parameters including central tendency (e.g. means) or other basic estimates (e.g. regression coefficient) AND variation (e.g. standard deviation) or associated estimates of uncertainty (e.g. confidence intervals)
☐	☒	For null hypothesis testing, the test statistic (e.g. *F*, *t*, *r*) with confidence intervals, effect sizes, degrees of freedom and *P* value noted<br>*Give P values as exact values whenever suitable.*
☒	☐	For Bayesian analysis, information on the choice of priors and Markov chain Monte Carlo settings
☒	☐	For hierarchical and complex designs, identification of the appropriate level for tests and full reporting of outcomes
☐	☒	Estimates of effect sizes (e.g. Cohen's *d*, Pearson's *r*), indicating how they were calculated

Our web collection on statistics for biologists contains articles on many of the points above.

Software and code

Policy information about availability of computer code

Data collection	Survey data were collected using a combination of REDCap and a custom database created by the Institute for Clinical & Translational Research at the Baylor College of Medicine
Data analysis	Analytic code for these analyses was written for R version 4.0.3, including the mice package version 3.8.0. This code is available from the corresponding author on reasonable request.

For manuscripts utilizing custom algorithms or software that are central to the research but not yet described in published literature, software must be made available to editors and reviewers. We strongly encourage code deposition in a community repository (e.g. GitHub). See the Nature Portfolio guidelines for submitting code & software for further information.

Data

Policy information about availability of data

All manuscripts must include a data availability statement. This statement should provide the following information, where applicable:

- Accession codes, unique identifiers, or web links for publicly available datasets
- A description of any restrictions on data availability
- For clinical datasets or third party data, please ensure that the statement adheres to our policy

The datasets generated and/or analyzed during the current study are not publicly available to protect the privacy of participants, but are available from the corresponding author on reasonable request.

Field-specific reporting

Please select the one below that is the best fit for your research. If you are not sure, read the appropriate sections before making your selection.

☐ Life sciences ☒ Behavioural & social sciences ☐ Ecological, evolutionary & environmental sciences

For a reference copy of the document with all sections, see nature.com/documents/nr-reporting-summary-flat.pdf

Behavioural & social sciences study design

All studies must disclose on these points even when the disclosure is negative.

Study description	The study was a pilot randomized controlled clinical trial.
Research sample	Provider participants were primary care physicians and cardiologists at Brigham and Women's Hospital. Patient participants were apparently-healthy primary care patients, ages 40-65 at recruitment, and cardiology patients with diagnoses of hypertrophic or dilated cardiomyopathy who were patients of participating physicians.
Sampling strategy	Participants were a convenience sample.
Data collection	Data were primarily participant reported outcomes. Physician data were collected via survey immediately after disclosure sessions. Participant data were collected via survey at enrollment, immediately post-disclosure, 6 weeks, and 6 months after disclosure sessions.
Timing	Participants were followed from 2012 to 2016.
Data exclusions	Analyses omitted four randomized patient-participants who did not have disclosure sessions, including two who died, one who withdrew due to concerns about potential genetic discrimination, and one who was lost to follow-up.
Non-participation	Non-participants are summarized in Robinson JO, Carroll TM, Feuerman LZ, et al. Participants and study decliners' perspectives about the risks of participating in a clinical trial of whole genome sequencing. J Empir Res Hum Res Ethics. 2016;11(1):21-30.
Randomization	We randomized 100 apparently-healthy primary care participants and 100 cardiology participants to receive a review of their family histories of disease, either alone or in addition to GS analyses.

Reporting for specific materials, systems and methods

We require information from authors about some types of materials, experimental systems and methods used in many studies. Here, indicate whether each material, system or method listed is relevant to your study. If you are not sure if a list item applies to your research, read the appropriate section before selecting a response.

Materials & experimental systems

n/a	Involved in the study	
☒	☐	Antibodies
☒	☐	Eukaryotic cell lines
☒	☐	Palaeontology and archaeology
☒	☐	Animals and other organisms
☐	☒	Human research participants
☐	☒	Clinical data
☒	☐	Dual use research of concern

Methods

n/a	Involved in the study	
☒	☐	ChIP-seq
☒	☐	Flow cytometry
☒	☐	MRI-based neuroimaging

Human research participants

Policy information about studies involving human research participants

Population characteristics	Provider participants were primary care physicians and cardiologists at Brigham and Women's Hospital. Patient participants were apparently-healthy primary care patients, ages 40-65 at recruitment, and cardiology patients with diagnoses of hypertrophic or dilated cardiomyopathy who were patients of participating physicians.
Recruitment	Physicians were recruited by interpersonal connections and via study presentations. They then provided MedSeq project staff with names of eligible patients to approach using letters and via telephone. Eligible primary care patients were generally healthy adults aged 40-65 years when approached for recruitment. Eligible cardiology patients were adults of any age with diagnoses of hypertrophic or dilated cardiomyopathy (HCM/DCM), who had previously received prior or concurrent panel-based genetic testing. find a genetic explanation for their condition. Exclusion criteria included prior diagnosis of diabetes, ongoing pregnancies, and lack of English fluency. Primary care patients with cardiovascular disease were also excluded.

Ethics oversight	The Mass General Brigham (formerly Partners HealthCare) Human Research Committee and the Baylor College of Medicine Institutional Review board approved the entire study.

Note that full information on the approval of the study protocol must also be provided in the manuscript.

Clinical data

Policy information about clinical studies

All manuscripts should comply with the ICMJE guidelines for publication of clinical research and a completed CONSORT checklist must be included with all submissions.

Clinical trial registration	NCT01736566
Study protocol	Published at as Vassy J, Lautenbach D, McLaughlin H, et al. The MedSeq Project: a randomized trial of integrating whole genome sequencing into clinical medicine. Trials. 2014;15(1):85.
Data collection	Data reported here were provided via web-administered surveys by participants.
Outcomes	Analyses reflected hypotheses per the funded grant. Measures for the pilot RCT were created by the study team and published Vassy J, Lautenbach D, McLaughlin H, et al. The MedSeq Project: a randomized trial of integrating whole genome sequencing into clinical medicine. Trials. 2014;15(1):85.
